# Impact of prolonged chronic social isolation stress on behavior and multifractal complexity of metabolic rate in *Octodon degus*

**DOI:** 10.3389/fnbeh.2023.1239157

**Published:** 2023-10-20

**Authors:** Grisel Cavieres, Francisco Bozinovic, José Miguel Bogdanovich, Daniela S. Rivera

**Affiliations:** ^1^Departamento de Zoología, Facultad de Ciencias Naturales y Oceanográficas, Universidad de Concepción, Concepción, Chile; ^2^Center of Applied Ecology and Sustainability (CAPES), Pontificia Universidad Católica de Chile, Santiago, Chile; ^3^Departamento de Ecología, Facultad de Ciencias Biológicas, Pontificia Universidad Católica de Chile, Santiago, Chile; ^4^GEMA Center for Genomics, Ecology and Environment, Facultad de Ciencias, Ingeniería y Tecnología, Universidad Mayor, Santiago, Chile

**Keywords:** behavior, social isolation stress, multifractality, metabolic rate, *Octodon degus*

## Abstract

Social interaction can improve animal performance through the prevention of stress-related events, the provision of security, and the enhancement of reproductive output and survival. We investigated the effects of prolonged chronic social isolation stress on behavioral, cognitive, and physiological performance in the social, long-lived rodent *Octodon degus*. Degu pups were separated into two social stress treatments: control (CTRL) and chronically isolated (CI) individuals from post-natal and post-weaning until adulthood. We quantified anxiety-like behavior and cognitive performance with a battery of behavioral tests. Additionally, we measured their basal metabolic rate (BMR) and analyzed the multifractal properties of the oxygen consumption time series using Multifractal Detrended Fluctuation Analysis, a well-known method for assessing the fractal characteristics of biological signals. Our results showed that CI induced a significant increase in anxiety-like behaviors and led to a reduction in social and working memory in male degus. In addition, CI-treated degus reduced the multifractal complexity of BMR compared to CTRL, which implies a decrease in the ability to respond to environmental stressors and, as a result, an unhealthy state. In contrast, we did not observe significant effects of social stress on BMR. Multivariate analyses showed a clear separation of behavior and physiological variables into two clusters, corresponding to CI and CTRL degus. This study provides novel insights into the effects of prolonged chronic social isolation stress on behavior, cognitive performance, and metabolic complexity in this rodent animal model. To the best of our knowledge, it is the first study to integrate cognitive-behavioral performance and multifractal dynamics of a physiological signal in response to prolonged social isolation. These findings highlight the importance of social interactions for the well-being and overall performance of social animals.

## Introduction

Interactions among conspecifics play a crucial role in the social structure of many species, contributing to the maintenance of their health, reproductive output, and survival (Neumann, 2009; Buwalda et al., 2013). Furthermore, the positive effects of social interaction attributed to social buffering serve to promote and/or maintain social cohesion (Hennessy et al., 2009). In this context, maternal presence constitutes the primary partner with which an animal forms a strong affiliative relationship, contributing to the formation and maintenance of filial attachment or pair-bonds throughout its lifespan (Shionoya et al., 2007; Hennessy et al., 2009). Social interactions are not only essential for many life processes, such as communal rearing of young and cooperation within a group (Neumann, 2009; Blumstein et al., 2010; Ebensperger et al., 2012), but they also play a protective role against stress-related events such as depredation, resource acquisition or defense, and social thermoregulation (Ebensperger, 2001; Gilbert et al., 2009).

Recently, it has been emphasized that stress is not only a “fight-or-flight” response to environmental threats, but also an ongoing process by which the body and brain adapt to daily experiences, including social environment, physical activity, and hazardous environments (McEwen and Akil, 2020). The physiological process of achieving stability or homeostasis through the production of mediators, such as hormones of the hypothalamus-pituitary–adrenal (HPA) axis, in response to challenges of daily life is known in stress biology as “allostasis” (McEwen and Wingfield, 2003). As life stressors persist, the adaptation process can be costly for organisms, triggering neurological and psychiatric disorders, including anxiety, emotional disturbances, and aggressive states, as well as atrophy of brain structures, cardiovascular disease, and other systemic disorders (McEwen and Wingfield, 2003; McEwen and Akil, 2020). “Allostatic load” is the price the body pays for adaptation to adverse situations (McEwen and Wingfield, 2003).

Disruptions to the social environment (e.g., social isolation or social instability) are associated with a range of physiological, neuroendocrine, and behavioral dysfunctions in both humans and nonhuman species that can lead to increased mortality and/or reduced lifespan (Beery and Kaufer, 2015; Mumtaz et al., 2018). These effects vary depending on species, sex, age, life stage, duration of exposure, and individual perceptions of the stressors (Lapiz et al., 2003; Neumann, 2009; Mumtaz et al., 2018; Rivera et al., 2020). Interestingly, among highly social mammals such as humans and primates, positive social relationships lead to substantial mental and physical health benefits in stress situations, reducing the risk of mortality (Taylor, 2006; Beery and Kaufer, 2015). Animal models, such as rodents, demonstrated that a lack of social interaction has behavioral effects, including impaired recognition memory, reversal learning, and an inability to make decisions (Zhao et al., 2009; Mumtaz et al., 2018), as well as physiological effects, such as an increase in chronic sympathetic tone, oxidative stress, and activation of the HPA axis, and a reduction in inflammatory control (McEwen and Wingfield, 2003; Cacioppo et al., 2011). Neurobiological consequences were also observed, including changes in the neuronal structure and brain activity (Lapiz et al., 2003; Westenbroek et al., 2004; Schoenfeld and Gould, 2012). Given these pervasive effects, more sophisticated assessment techniques using multidimensional measures are needed to test the impact of social isolation stress on animal performance.

Biological systems exhibit great complexity. This complexity results from the interactions of multiple structural units and regulatory feedback loops that enable living organisms to effectively cope with the diverse stresses and challenges of daily life (Lipsitz, 2004). The stated variables of healthy organisms exhibit complex fluctuations characterized by long-range power-law correlations that arise through multifractal cascades operating over a wide range of timescales (Goldberger et al., 2002). The breakdown of this multi-scale complexity has been recognized as a hallmark of pathologies and aging (Hausdorff et al., 2001; Costa et al., 2008). In this context, multifractal analysis of biosignals has become a useful tool in biology and biomedicine. It allows us to understand the abnormalities that lead to many pathologies and to provide simple numerical descriptions of complex biological systems and their diseases, including COVID-19 (Li, 2021), Parkinson’s disease (Marmelat and Meidinger, 2019), obstructive sleep apnea (Zhang et al., 2009), and cardiovascular disease (Peskin and McQueen, 1994). Thus, this approach is emerging as a powerful diagnostic tool for determining physiological health, and it holds great promise for improving our understanding of the physiological consequences of stress.

Animal studies on the effects of social isolation stress are a valuable counterpart to human studies. Due to the potential risk of damaging effects, randomized and experimental manipulations of social isolation in humans are limited in intensity and duration. In addition, the last few years have seen increased interest in the effects of long-term social isolation, mostly associated with the quarantines imposed during the COVID-19 pandemic. Thus, longitudinal studies in animals are key to elucidating anxiety-related effects on individuals’ behavior and physiology due to isolation-derived stress. Here, we selected the degu (*Octodon degus*) as a suitable model for studying long-term social-affective biological behavior since this rodent shares many physiological and behavioral characteristics with humans, including the ability to form social bonds and extended social group families (Silk, 2007; Colonnello et al., 2011; Rivera et al., 2016). The main advantage of using the degu as an animal model is the degus’ relatively long lifespan under captive conditions [7–8 years, Lee (2004)], which allows researchers to assess the effects of natural aging and age-related diseases. Aging degu brains naturally develop amyloid plaques and tau phosphorylation, two hallmarks of Alzheimer’s disease (Inestrosa et al., 2005; Tarragon et al., 2013; Hurley et al., 2018). Furthermore, during the aging process, degus exhibit cognitive decline and memory impairment similar to that seen in humans with dementia (Ardiles et al., 2012; Rivera et al., 2021b). Recently, we reported that long-term social isolation not only affects stress physiology by impairing the negative feedback loop of the HPA axis, resulting in prolonged elevated blood cortisol levels following a stressor, but also has effects on behavioral, functional, and molecular traits related to spatial and social memory-related tasks in degus (Rivera et al., 2020, 2021a). This study aimed to investigate cognitive and physiological performance and multifractal properties of metabolic time series r(VO_2_) in degus exposed to prolonged social isolation stress. To the best of our knowledge, this is the first study to examine the fractal properties of time series of r(VO_2_) and cognitive performance in a long-lived animal model exposed to prolonged social isolation stress, providing novel insights into the impact of such a stressor on physiological and cognitive functions in a social mammal.

## Materials and methods

Laboratory pregnant female degus obtained from our colony at the Faculty of Biological Sciences, Pontificia Universidad Catόlica de Chile were kept in pairs and housed in clear acrylic terrariums (length × height × depth: 50 × 35 × 23 cm) with hardwood chip bedding. Each cage contained one nestbox made of clear acrylic (22 × 12 × 15 cm). We checked for litters daily, and the day of birth was defined as postnatal day (PND) 0. To avoid litter differential parental effects within a litter, the whole litter was randomly assigned to one of the following rearing conditions: (i) unstressed controls (CTRL, *N* = 12) in which the male litters were left undisturbed with their family. The siblings remained together until PND 90, and thereafter they were raised as male-matched groups of three siblings from PND 91 until the end of the experiment; (ii) Chronically isolated individuals (CI, *N* = 12) in which the male pups were removed from their mothers and kept individually in small opaque cages for 1 h daily (between 09:00 a.m. and 12:00 p.m.) from PND 1 to PND 35 (day of weaning). During separation, the pups had acoustic and olfactory but no visual and social contact with their siblings or mother. From PND 36 until the end of the experiment, they were individually housed in standard rodent cages with no physical contact with conspecifics (though olfactory, acoustic, and some visual contact could not be fully prevented).

All animals were kept in a ventilated room and exposed to a 12 L:12D setting and ambient temperature (yearly minimum = 13.4 ± 0.2°C; yearly maximum = 24.9 ± 0.2°C) and were fed a standard rabbit commercial pellet diet (Champion, Santiago, Chile) and *ad libitum* water. All animal protocols followed the guidelines of the National Institutes of Health (NIH, Baltimore, MD, USA). All efforts were made to minimize animal suffering and to reduce the number of animals used.

### Behavioral observations

At the end of the 22-month period, a total of 24 males (*n* = 12 for CTRL and *n* = 12 for CI) were subjected to five behavioral trials, as described in detail below. To minimize the effects of behavioral experience on the results, the experiments were conducted in the order from the least to the more intrusive, running one test per day. The order of experiments was as follows: (i) open field test, (ii) “novel object” open field test, (iii) light–dark box test, (iv) social interaction test, and (v) novel local and novel object recognition test. All behavioral tests were conducted during the animals’ active phase between 09:00 and 16:00 h at an average temperature of 20.0°C. At the end of each session, the animals were returned to their home cages, and the area was wiped clean with a 70% ethanol solution.

### Open field test

Animals were observed for 5 min in the open field test arena that consisted of a white plexiglas box (length × height × depth: 100 × 100 × 100 cm). The frequency of total crossings and “central crossings” (with a four-paw criterion) were scored (Rivera et al., 2018). In addition, the percentage of time in corners, in the middle arena, speed, and total length were assessed (Rivera et al., 2018).

### The “novel object” open field test

This test measures the animal’s response toward a novel object placed in the center of the open field. Specifically, an emotionally relaxed degu would approach the novel object often to investigate, whereas an anxious, hyperactive animal would be much less keen to explore the unfamiliar object (Rivera et al., 2021a). Subjects underwent the “novel object” open field test the day following the conventional open field. In the same arena as the open field test, animals were observed for 5 min. The total time spent by the animal exploring this distinct novel stimulus was recorded. In addition, the percentage of time spent in corners and the middle arena, as well as the speed and total distance traveled, was assessed (Rivera et al., 2021a). At the end of each session, the animals were returned to their home cages, and the area was wiped clean with a 70% ethanol solution.

### Light–dark box test

The light–dark box test is a frequently used test for anxiety-like behavior (Crawley and Goodwin, 1980). The Light–dark box consisted of a cage made of Plexiglas box, divided into two equalized brightly illuminated and fully black sections (length × height × depth: 21 × 21 × 21 cm). Both sections were connected by a 7 × 7 cm opening at the floor level. The white (light section) remained uncovered during the test and was illuminated by a 24 V–10 W bulb. Whereas the black (dark section) was covered by a light-proof lid without appreciable illumination. For this test, we followed the light–dark box protocol used for degus by Popović et al. (2009). Briefly, the animal was placed in the light section, facing away from the entrance to the dark section, and allowed to explore the apparatus freely for 10 min. The behavior recorded during the test included: latency to the dark box (i.e., the time it took the animals to enter the dark box) (Costall et al., 1989), number of transitions (i.e., number of times the animals crossed from the light to the dark box, and vice versa) and total duration in the light box (i.e., the amount of time spent by the animal in the light box). At the end of each session, the animals were returned to their home cages, and the apparatus was wiped clean with a 70% ethanol solution.

### Social interaction test

We used the three-chamber test to assess (i) social affiliation/motivation by comparing the time degus spent interacting with an empty wire cage versus one containing a novel degu and (ii) social memory and preference for social novelty by measuring the time degus spent interacting with an unfamiliar (novel) versus a familiar degu. The open field arena was subdivided into three equal compartments using transparent Plexiglas walls, each with a small opening (diameter 2.8 cm), allowing access into each compartment. The degus to be used as social partners were male sex matched, unfamiliar, and unrelated. The test comprised three 20-min sessions. The social test was performed following the protocol previously described by Rivera et al. (2018). Briefly, three phases were performed in the following order: Phase 1 or Habituation: the test animal was placed in the middle compartment and allowed to explore the apparatus. Phase 2 or Session 1 (“Partner I”): the test animal was returned to the middle compartment. Simultaneously, the first social partner (a sex-matched, unfamiliar, and unrelated degus) was placed inside a wire containment cup located in one of the side chambers. The test degu was then free to interact with the social partner or with the empty cup. At the end of Session 1, the test animal was returned to his home cage for 1 h, and the area was wiped clean with a 70% ethanol solution to remove odors. Phase 3 or Session 2 (“Partner II”): The test animal was again returned to the middle compartment, and a second unfamiliar, unrelated degus was placed inside an identical wire containment cup in the opposite side chamber, which had been empty during Session 1. In this part, the test animal is free to choose between the first, already-investigated, unfamiliar degus (Partner I), and a novel unfamiliar animal (Partner II). As measurements of social interaction, we recorded the time spent exploring the partner (i.e., the time where the test animal spent touching the containment cup housing or not housing the new partner for each chamber individually, with the forepaw or nose). To evaluate differences in social affiliation/motivation and social memory, we calculated the recognition index (RI). The RI for Session 1 was defined as the quotient of the time the degus spent with Partner I divided by the sum of the time spent with Partner I and the empty cup. For Session 2, the RI was calculated as the time spent with Partner II divided by the sum of the time spent with Partners I and II. A RI ≤ 0.50 indicates that degus have an absence of social affiliation/motivation during Session 1 and an absence of social memory during Session 2 (Rivera et al., 2021a).

### Novel local and novel object recognition test

The Novel Local/Novel Object Recognition test is a double test used to evaluate cognition, particularly working memory and attention, but also can be used to test the preference for novelty in rodents (Popović et al., 2009). The test arena used an open box (length × height × depth: 63 × 40 × 30 cm) made of white Plexiglas. For this test, we followed the object recognition protocol previously used in degus (Rivera et al., 2018). Briefly, animals were exposed individually to a 10-min familiarization assay and then subjected to two consecutive 5-min assays separated by a 1-h inter-trial interval. Session 1 (Familiarization): two objects (“Object A” and “Object B”) were placed in the corners of the home cage, and the animal was allowed to explore them freely for 10 min. Following this period, objects were removed from the cage and wiped with a 70% ethanol solution and the test animal was returned to its home cage for 1 h. For Session 2 (“Novel location recognition” or NLR): one of the familiar objects (Object B) was moved to an adjacent unoccupied corner. The test animal was then free to interact with the objects during 5 min. Following this period, objects were removed from the cage and wiped with a 70% ethanol solution and the test animal was returned to its home cage for 1 h. For Session 3 (“Novel Object Recognition” or NOR): the familiar Object B was replaced by a different, but similar, object. We recorded the familiarization and testing times, and the time spent exploring each object. “Exploration” time was defined as approaching to within 1–3 cm of the object. To quantify NLR and NOR, a Recognition Index (RI) was calculated as the time spent with object B divided by the sum of the time spent with object A and object B. A normal and unstressed rodent would tend to explore the novel object more than the familiar one. Then, a RI above 0.5 indicates greater investigation of the novel location or object (Rivera et al., 2020). In all cases, a digital video camera (LifeCam Studio Full HD, Microsoft Corp, Redmond, WA, USA) was mounted above the test arena, and the performance of each animal was monitored with image tracking software (HVS Image, Hampton, UK).

### Physiological traits

#### Basal metabolic rate

We measured the rate of oxygen consumption (VO_2_) in 24 male degus post-absorptive (six-hour fasted), resting in the inactive phase (19:00 h–07:00 h), using standard flow-through respirometry system. Animals were kept in 2000 mL steel metabolic chambers with a wire-mesh grid that allowed excreta to fall into a tray containing mineral oil, thus trapping water from this source. Oxygen consumption was measured using a computerizing, open flow respirometry system calibrated with a known mix of oxygen (20%) and nitrogen (80%) certified for chromatography (INDURA, Chile). Measurements were randomized and performed at ambient temperature of 30.0 ± 0.5°C, which is well within the thermoneutral zone for this species (Bozinovic and Rosenmann, 1988). Before passing through the metabolic chamber, air passed through CO_2_ absorbent granules of Baralyme^®^ and Drierite^®^ at 800 mL min^−1^ from a mass flow controller (Sierra Instruments, Monterey, CA, USA). The mass flow meter was calibrated monthly with a volumetric flowmeter. After passing through metabolic chamber, the excurrent air passed through columns of CO_2_ absorbent granules of Baralyme and Drierite before passing through an O_2_-analyzer (model FC-10A, Sable Systems). Output from the oxygen analyzers (%) was digitized using a Universal Interface II and recorded on a personal computer using EXPEDATA data acquisition software (Sable Systems, Las Vegas, USA). Since CO_2_ was scrubbed before entering the O_2_ analyzer, oxygen consumption was calculated as:


(1) $$VO_{2}=\left[FR\times 60\times \left({FIO}_{2}-{FEO}_{2}\right)\right]/\left(1-{FIO}_{2}\right)$$

where FR is the flow rate in ml min^−1^ after standard temperature and pressure correction, and FI and FE are the fractional concentrations of O_2_ entering and leaving the metabolic chamber, respectively. The oxygen consumption was monitored allowing us to obtain time series r(VO_2_) records at periodic intervals of *t* = 1 s. We estimated basal metabolic rate (BMR) from the VO_2_ records over 10 min period after a steady state was reached. Body mass (Mb) was measured prior to metabolic measurements using an electronic balance (±0.1 g), and rectal body temperature (Tb) was recorded at the end of each measurement using a Digi-Sense copper constantan thermocouple.

#### Multifractality analyses of metabolic rate: proprieties and causality

We analyzed the multifractal properties of the oxygen consumption time series in degus reared under CTRL and CI treatments using the Multifractal Detrended Fluctuation Analysis (MF-DFA). Furthermore, we investigated the causes of the observed multifractality in the time series (Kantelhardt et al., 2002; Labra et al., 2016; Li, 2021).

MF-DFA calculates a profile or accumulated data series from every metabolic dataset $y_{i}=\sum r\left(VO_{2},i\right)\text{,}$ for all samples i=1 to *N*. The profile is subdivided into *Ns* non-overlapping segments of length *s*. For every segment v, the local trend was fitted by a polynomial of order 1, 2, or 3. The resulting variance is then raised to the q/2th power${\left[\sigma^{2}\left(v,s\right)\right]}^{q/2}$ and a generalized fluctuation function $F_{q}\left(s\right)$ is calculated by averaging all the variances across all segments of scale s (equation 2) (Kantelhardt et al., 2002; Ludescher et al., 2011)


(2) $$F_{q}\left(s\right)={\left\{\frac{1}{N_{s}}\overset{N_{s}}{\underset{v=1}{\sum }}{\left[\sigma^{2}\left(v,s\right)\right]}^{\frac{q}{2}}\right\}}^{\frac{1}{q}}$$

In a multifractal signal, the fluctuation function (*F_q_*) scales within some range of *s* according to power law: $F_{q}\left(s\right)\sim s^{h\left(q\right)}$ . The scaling exponent *h*(*q*), called generalized exponent Hurst, is obtained as slope of regression between ln(*Fq(s)*) and ln(*s*). The *h*(*q*) exponent defines the fractal structure of the time series by how fast the *Fq(s)* grows with increasing scale *s* (Li, 2021). The dependence of *h*(*q*) on *q* is used to examine the multifractal properties of a time series (Zhang et al., 2019). For a monofractal time series, *h(q)* is independent of *q* (i.e., the small and large fluctuations scale differently), while for multifractal time series, *h*(*q*) is a decreasing function of *q*. When *q* = 2, the *h(q)* value is identic to the well-known Hurst exponent (*H*). H values range between 0 and 1; when *H* = 0.5, the time series are uncorrelated and random, for *H* > 0.5 indicates a long-range correlation structure with memory or persistence; finally, values of *H* < 0.5 indicates that the series has a short-range correlation structure (i.e., anti-memory, or anti persistence) (Zhang et al., 2019). Also, the range of *h(q)*, *∆h = h(q_min_)-h(q_max_)* indicates the extent to which the series is multifractal. High *Δh* values imply strong multifractality. Furthermore, *h(q)* can be directly related to the classical multifractal scaling Renyi exponents *τ(q)* defined by *τ(q)* = *qh(q)* – 1, and *h(q)* = (*τ(q)* + 1)/*q* (Koscielny-Bunde et al., 2006; Suárez-García and Gómez-Ullate, 2014). In the MF-DFA, the singularity spectrum is estimated from the relationship between generalized exponent *h(q)* and scaling exponent function *τ(q)* after the Legendre transformation (equation 3)


(3) $$f\left(\alpha \right)=q\alpha -\tau \left(q\right)=q\left[\alpha -h\left(q\right)\right]+1$$

Multifractal spectrum *f (α)* describes the fractal dimension of the ensemble formed by all the points that share the same singularity exponent α. Fractal dimension *f (α)* ∼ α is shaped like a single-peaked bell. The multifractal spectrum width is the difference between the maximum and minimum singularity exponent, *Δα = α_max_ − α_min_*, which reveals how strong the multifractality of the time series is. The type of the extreme fluctuation rate occurring with a higher probability can be estimated using $\Delta f=f\left(\alpha_{\text{min}}\right)-f\left(\alpha_{\text{max}}\right)$ which is the difference between the fractal dimension of the maximum probability subset and that of the minimum probability subset. When Δf > 0, the maximal fluctuation rate occurs with a higher possibility than that of the minimal fluctuation rate and vice versa (Li, 2021).

Two sources of multifractality in time series have been proposed: fat tails and long-range temporal correlations. These can be distinguished by shuffling and surrogate procedures and comparing the multifractal characteristics with the original time series (Rak and Zięba, 2015). The shuffling procedure removes any temporal correlations to generate a random permutation of the array elements of the time series. If the multifractal feature remains after the shuffling procedure, the long-range correlation dominates the multifractality in the original series. The proportion between the shuffle series and original series (Shuffle ratio = ∆α_shuff_/∆α_original_) indicates the contribution of the long-range correlation to multifractal strength. The surrogate procedure randomizes all-time series using an amplitude-adjusted Fourier transform algorithm (AAFT) (Schreiber and Schmitz, 1996, 2000). The ratio between the surrogate and original series (Surrogate ratio = ∆α_surr_/∆α_original_) indicates the proportion of the fat-tailed probability distribution (Agbazo et al., 2019). The shuffling and surrogate methods can be combined to understand the sources of the empirically estimated apparent multifractality.

## Statistical analysis

The assumptions of normality and homoscedasticity were confirmed using Shapiro–Wilk and Levene’s tests, respectively. For univariate analyses, we used *t*-tests to analyze the effect of stress treatment groups on male degus across each variable of the behavioral test. All behavioral data are presented as the mean ± SEM. In social interaction and NLR-NOR tests, the IR were analyzed. Also, we assess the effects of social interaction on BMR using a linear model with following variables: BMR * Mb. To assess the multifractal properties of the oxygen consumption time series for CTRL and CI groups, we considered six parameters: the Hurt exponent (*H*) and the variability of *h(q)* (*∆h(q)*), which were used to characterize the dependence profile of *h*(*q*) on *q*. For the singularity spectrum, we used the width of spectrum (*∆a*), amplitude of spectrum (*∆f*), the asymmetry parameter (B), and dominant exponent (*a_0_*). To estimate the parameters of multifractal properties, we used the package *fractal*. We used a *t*-test to compare these parameters between the treatments.

To analyze the effects of social stress on the physiology and behavior of the degus in an integrated manner, we carried out a Principal coordinate analysis (PCoA) and a multivariate non-parametric analysis of dissimilarities (PERMANOVA) using a Manhattan dissimilarity matrix. Pairwise distances were calculated for all variables (behavior-cognitive related traits in Table 1, and physiological traits BMR, Mb, Table 2), and the PCoA coordenates used to test differences between the CTRL and CI groups. The PERMANOVA was implemented in the *Adonis2* function within the *vegan* R package.^1^

**Table 1:** Behavioral measurements recorded in the open field, the “novel object” open field, the light–dark box, the social interaction and the Novel Local and Novel Object Recognition (NLR/NOR) tests.

	Stress treatment	
Behavioral observations	CTRL	CI
*Open field*		
% time in the central zone	21.15 ± 2.17	15.01 ± 2.28
% time in corners	35.85 ± 1.67	42.80 ± 4.70
Number of central crossings	9.00 ± 1.11	8.42 ± 1.42
Total distance moved [m]	39.26 ± 3.03	42.76 ± 4.64
Average speed [m/s]	0.13 ± 0.01	0.14 ± 0.01
*“Novel object” open field test*		
Time with the novel object [s]	21.74 ± 3.66	7.86 ± 2.75**
Number of entries into central zone	7.50 ± 0.89	3.00 ± 0.99**
% time spends in the central zone	28.81 ± 4.82	13.28 ± 3.34*
% time in the corners	37.36 ± 5.53	60.08 ± 7.05*
Total distance moved [m]	38.45 ± 4.09	33.85 ± 6.48
Average speed [m/s]	0.12 ± 0.01	0.11 ± 0.02
*Light–dark box test*		
Number of light–dark box transitions	30.83 ± 2.83	18.75 ± 2.25**
Time in the light box [s]	314.6 ± 17.25	247.1 ± 33.65
Latency to enter to the dark compartment [s]	17.32 ± 2.91	44.89 ± 11.61*
*Social interaction test*		
RI session I	0.87 ± 0.02	0.75 ± 0.03**
Time spent with partner I [s]	210.2 ± 14.27	146.2 ± 5.13**
Time spent with empty cup [s]	30.12 ± 4.75	50.4 ± 7.54*
RI session II	0.63 ± 0.03	0.44 ± 0.03**
Time spent with partner II [s]	129.2 ± 18.68	96.36 ± 9.55
Time spent with partner I [s]	69.59 ± 6.34	121.2 ± 10.68**
*NLR/NOR Test*		
RI NLR	0.72 ± 0.05	0.47 ± 0.04**
Time with the moved object [s]	16.39 ± 1.85	15.22 ± 2.45
Time with familiar object [s]	6.71 ± 1.49	21.99 ± 4.78**
RI NOR	0.81 ± 0.03	0.51 ± 0.03**
Time with the new object [s]	34.84 ± 4.56	15.22 ± 1.97**
Time with familiar object [s]	8.43 ± 1.59	14.62 ± 2.29*

CTRL, control group (*n* = 12); CI, chronic isolation (*n* = 12). Values are means ± SEM, *p < 0.05 and **p < 0.001.

**Table 2:** Parameters used to characterize the multifractal properties of the time series and their origin.

	*H*	*∆h(q)*	*α_0_*	*∆α_orig_*	*∆f*	*∆α_shuff_*	*∆α_surr_*
CTRL	0.44 ± 0.03	0.49 ± 0.05	0.59 ± 0.03	0.72 ± 0.05	−0.01 ± 0.10	0.25 ± 0.17	0.65 ± 0.01
CI	0.61 ± 0.03	0.32 ± 0.03	0.66 ± 0.03	0.52 ± 0.04	0.004 ± 0.10	0.26 ± 0.02	0.58 ± 0.01
Test	−3.48**	2.90**	−1.31	2.91**	0.1		

*H*, Hurst exponent; *∆h(q)*, Variability Hurst exponent profile; *α_0_*, dominant exponent; *∆α_orig_*, spectrum width of the original signals; *∆f*, spectrum amplitude. The spectrum width (∆α*shuff* and ∆α*surr*) was calculated from the shuffling and surrogate procedures. The values are means ± SEM. **p < 0.01.

## Results

### Behavioral and cognitive performance

#### Open field test

To evaluate the general state of the animals, we performed the open field test. We measured the time that the animal spent in the central zone and in the corners of the arena, the number of central crossings, the speed, and the total distance traveled. There was no effect of stress treatment on any of these measures (all *p* > 0.05), suggesting that general behavior is unaffected by social isolation stress.

#### The “novel object” open field test

It is expected that animals without emotional stress will interact with a novel object more often than animals that are emotionally perturbed. CI animals spent significantly less time near the novel object (unpaired *t* test, df = 22, *t* = 3.03, *p* < 0.01; Table 1) and entered the central zone of the field significantly fewer times (unpaired *t* test, df = 22, *t* = 3.37, *p* < 0.01; Table 1) than CTRL animals. We also recorded significant differences in the percentage of time spent in the corners (unpaired *t* test, df = 22, *t* = −2.54, *p* = 0.02; Table 1) and in the retention time in the central zone of the arena (unpaired *t* test, df = 22, *t* = 2.65, *p* = 0.01; Table 1). However, there were no differences in the total distance moved (*p* = 0.56) or average speed (*p* = 0.54) between the two groups. These findings suggest that in both the open field test and the novel object open field test, CI degus exhibited anxiety-like behavior compared to the CTRL degus. Furthermore, we were able to set aside differences associated with locomotor activity (total distance and average speed remained unchanged across the test).

#### The light–dark box test

The *t*-test analysis revealed a significant difference in the number of light–dark box transitions in the two groups (unpaired *t* test, df = 22, *t* = 3.34, *p* < 0.01; Table 1), with degus in the CI group making fewer transitions between the two chambers than CTRL degus. Degus in the CI group spent less time in the light part of the box than those in the CTRL group (Table 1), although the difference was not statistically significant (*p* = 0.09). Furthermore, the latency to enter the dark compartment was prolonged in the CI group (unpaired *t* test, df = 22, *t* = −2.30, *p* < 0.01; Table 1). Taken together, these results suggest that CI degus showed anxiety-like behavior compared to CTRL degus.

#### Social interaction test

The analysis of the effects of prolonged chronic social isolation on the degus’ performance in the social interaction test, using RI as the dependent variable, indicated a significant difference between the two groups in session 1 (unpaired *t* test, df = 22, *t* = 3.43, *p* < 0.01) and session 2 (unpaired *t* test, df = 22, *t* = 3.87, *p* < 0.01; Table 1). During session 1, both CTRL and CI groups showed a clear and significant preference for the compartment with partner I (unpaired *t* test, df = 22, *t* = 2.81, *p* = 0.01) than the compartment with the empty cup (unpaired *t* test, df = 22, *t* = −2.11, *p* = 0.04; Table 1). During session 2, the CTRL degus showed a clear preference for the compartment containing the novel unfamiliar degu (partner II) compared to the CI degus (Table 1), although the difference was not statistically significant (*p* = 0.11). In contrast, CI degus spent more time interacting with the old unfamiliar degu than the CTRL group (unpaired *t* test, df = 22, *t* = −3.96, *p* < 0.01; Table 1). Taken together, these results indicated that prolonged social isolation stress impaired social affiliation/motivation, social memory, and preference for social novelty.

#### Novel local and novel object recognition test

Taking the RI as the dependent variable, the analysis of the effects of prolonged chronic social isolation measured across the NLR trial revealed a significant difference between the two groups (unpaired *t* test, df = 22, *t* = 3.91, *p* < 0.01; Table 1). During the NLR trial, both groups showed similar preference for the moved object (*p* = 0.70; Table 1), but the CI group showed a greater preference for the familiar object than the CTRL degus (unpaired *t* test, df = 22, *t* = −2.99, *p* < 0.01; Table 1). Similarly, we observed a significant difference between the RI of the two groups during the NOR assay (unpaired *t* test, df = 22, *t* = 7.32, *p* < 0.01; Table 1). In this trial, the CTRL degus showed a clear preference for the novel object compared to the CI group (unpaired *t* test, df = 22, *t* = 4.04, *p* < 0.01; Table 1). The *t*-test analysis revealed that the CI degus spent more time interacting with the familiar object (unpaired *t* test, df = 22, *t* = −2.07, *p* = 0.04; Table 1), suggesting a reduced predilection for novel experiences compared with the CTRL group.

### Physiological performance

#### BMR and multifractality analyses of metabolic rate

*BM*R was significantly influenced by Mb (*F*_(1,18)_ = 10.1, *p* = 0.005), but neither social stress (*F*_(1,18)_ = 0.84, *p* = 0.37) nor the interaction between Mb and social stress (*F*_(1,18)_ = 0.21, *p* = 0.64) had significant effect on BMR. Mb did not show differences between the CTRL and CI treatments (*F*_(1,20)_ = 0.01, *p* = 0.91).

**Figure 1:**
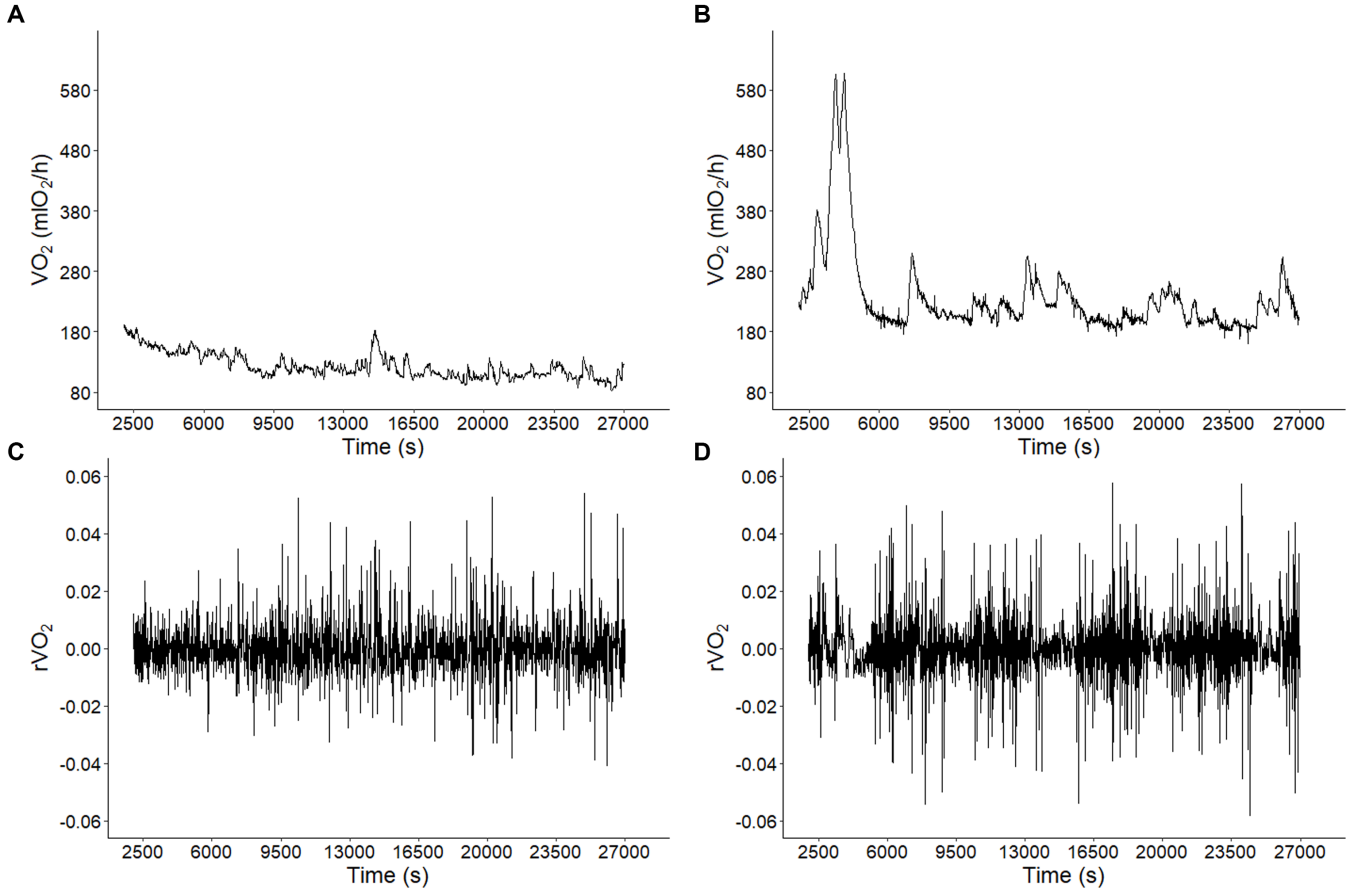
Long-term correlations of metabolic rate fluctuations in *Octodon degus*. Metabolic rate (VO_2_) time series for a representative animal from **(A)** control (CTRL) and **(B)** chronic social isolation stress (CI) groups during 9 h at 1 (s) intervals. Observed VO_2_ fluctuations r(VO_2_) = log_10_[VO_2_ (t + 1)/VO_2_ (t)] time series for data in A **(C)** and B **(D)**.

**Figure 2:**
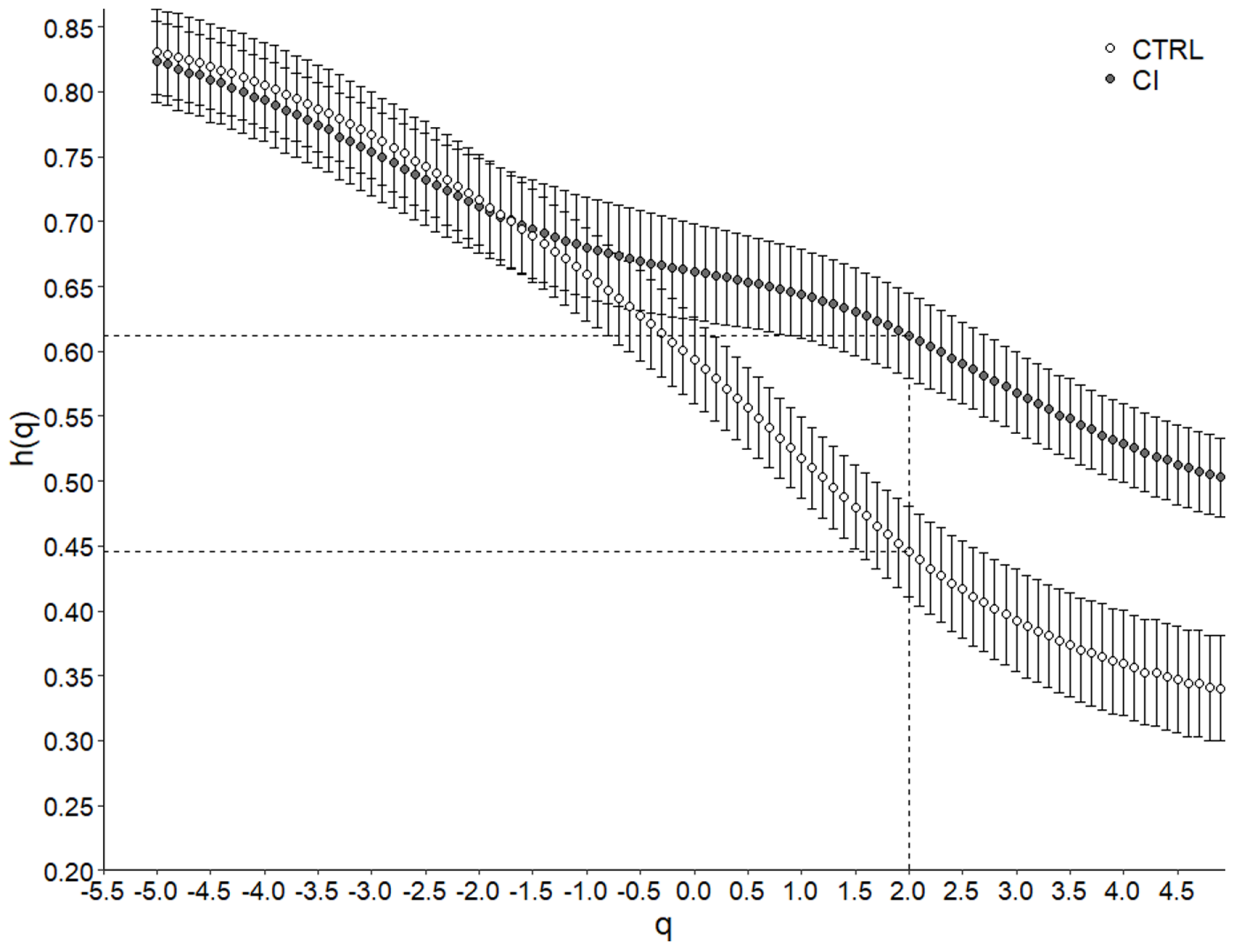
Multifractal Detrended Fluctuation Analysis of *Octodon degus* r(VO_2_) time series across stress treatments. Generalized Hurst exponent h(q) as a function of q for control (CTRL) and chronical isolated (CI) groups. Values correspond to the mean (±1 SEM). The dashed line indicates H or Hurst Exponent (when *q* = 2, h(q) = H) for each treatment.

Metabolic rate records in both CTRL and CI groups (Figures 1A,B) exhibited irregular non-stationary fluctuations. However, the detrended time series of r(VO_2_) in both groups (Figures 1C,D) revealed clusters of large fluctuations separated from clusters of small fluctuations. This pattern suggests the presence of long-term correlation or persistence in r(VO_2_) time series. We further supported these findings by finding a negative relationship between *h(q)* and *q* in both treatments (Figure 2). Interestingly, both groups showed a similar scaling behavior (i.e., small fluctuations) for *q* < 0. However, for *q* > 0, the profile of the CTRL group showed a higher slope, indicating that fluctuations of a larger size play a more significant role in the behavior of the time series of unstressed degus. The $\Delta h_{\left(q\right)}$ was 35% higher in the CTRL group compared to the CI group (Table 2; Figure 2), revealing a greater degree of multifractality for the CTRL group. We found a significantly higher Hurst exponent (*H*) value for the CI degus compared with the CTRL group (Table 2). This value exceeds 0.5, indicating that the rVO_2_ for CI degus behaves as a long-range correlated or persistent signal (i.e., higher predictability along the time series, Figure 2).

**Figure 3:**
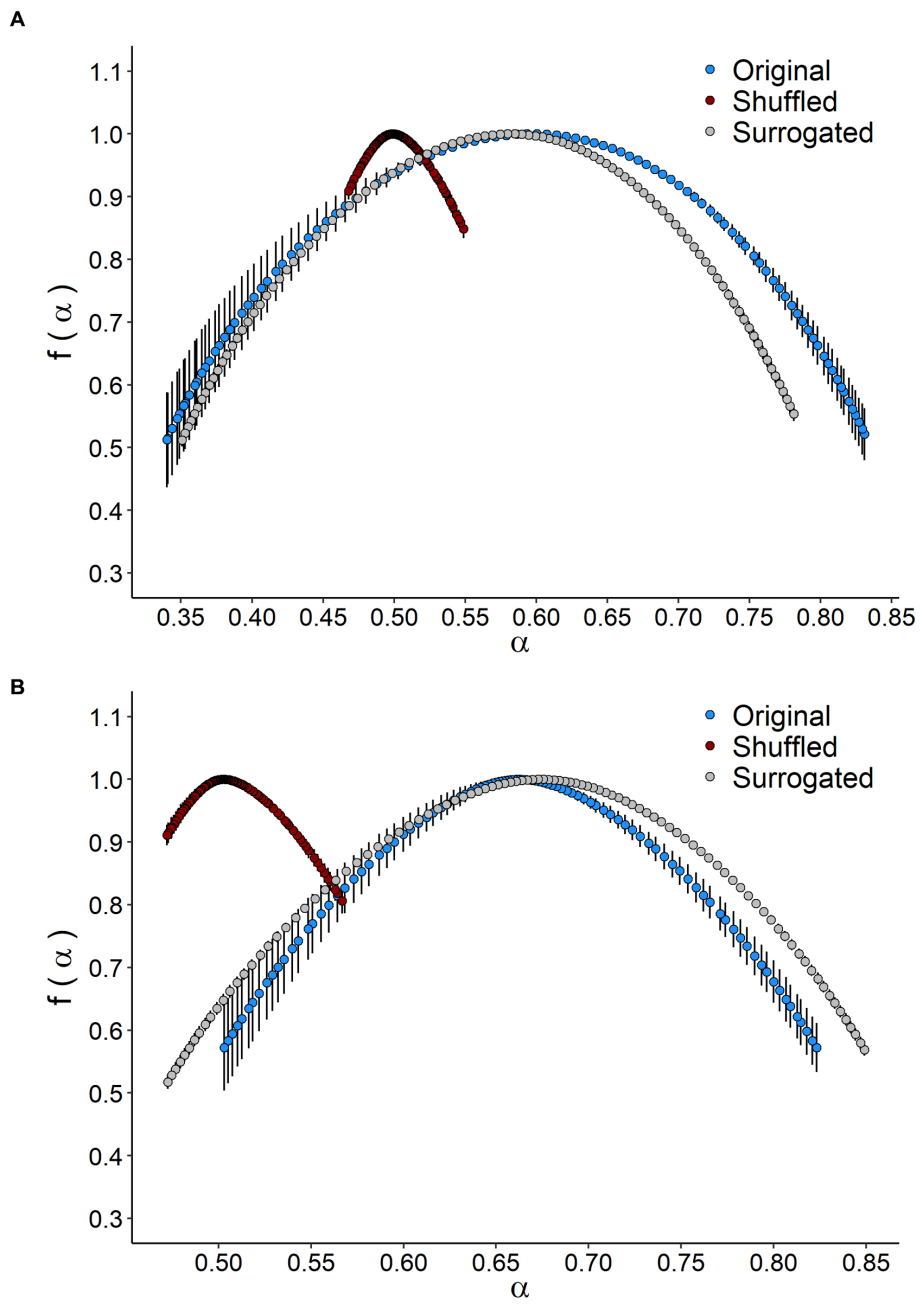
Singularity spectrum of VO_2_ time series in *Octodon degus*. The singularity spectrum *f(α)* as a function of a singularity strength *α* of original, shuffted, and surrogated series for **(A)** control (CTRL) and **(B)** chronic social isolation stress (CI) groups. Values correspond to the mean (±1 SEM).

The singularity spectrum of the multifractal time series shows a hump shape in both treatments (Figure 3). In the original time series, the spectrum width for the CI was significantly smaller than that observed for the CTRL group, indicating less complexity in multifractal behavior (Table 2; Figure 3). The singularity spectrum for both the CTRL and CI treatments exhibited a hump shape in the shuffle and surrogate curves (Figure 3). Additionally, the width of the singularity spectrum was similar for the original and surrogate curves but smaller for the shuffle series, indicating less heterogeneity in the time series. The shuffle ratios for the CTRL and CI treatments were 0.34 and 0.50, respectively, while the surrogate ratios were 0.89 and 1.11 (see Table 2). These results suggest that long-range correlations between small and large fluctuations dominate the signals.

**Figure 4:**
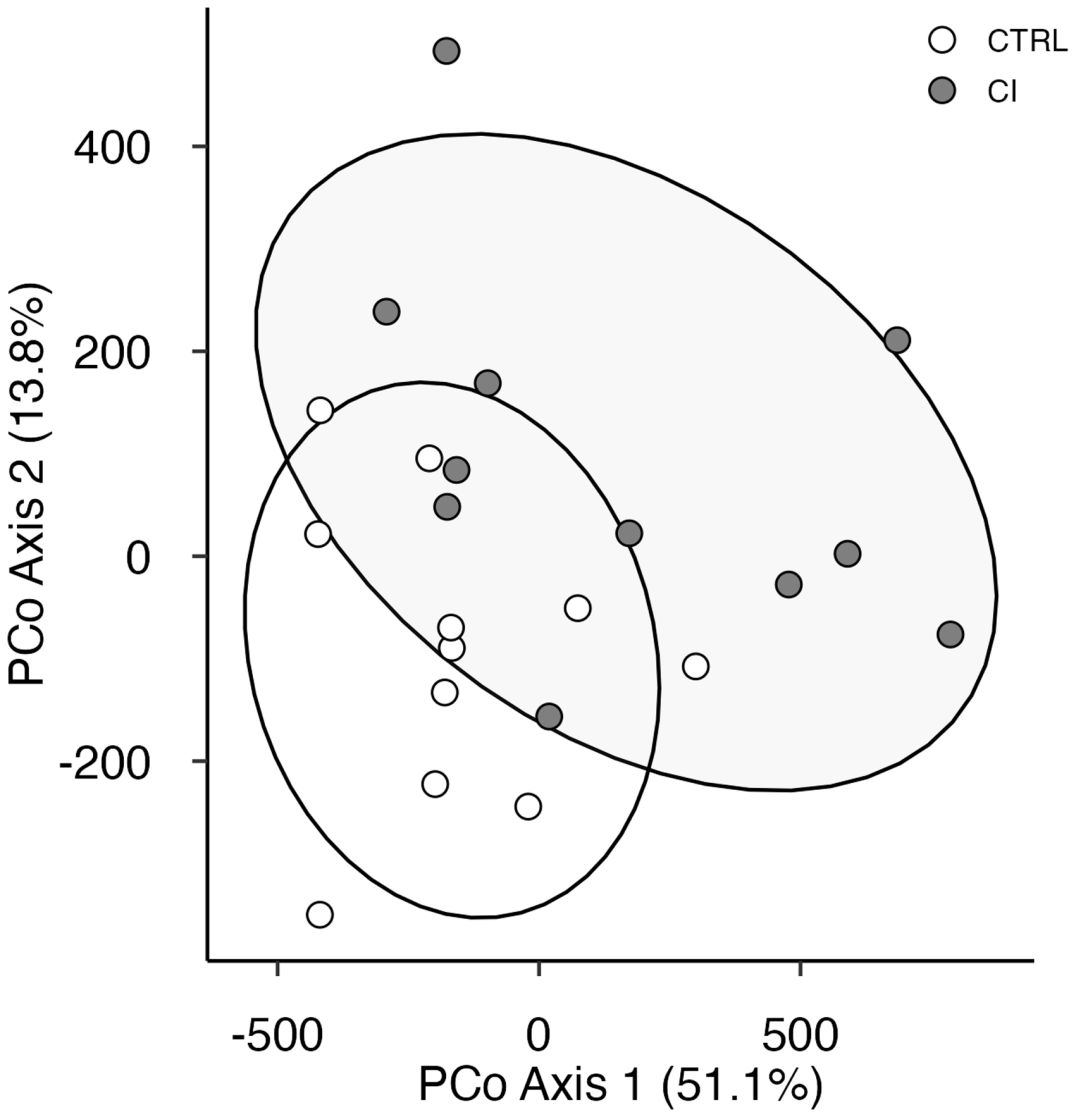
Principal coordinate analysis plot of behavioral and physiological variables in *Octodon degus*. Using Manhattan distances for control (CTRL, open circles) and chronic social isolation stress (CI, gray circles) groups.

Principal coordinates analysis revealed that the 26 variables in the model could be grouped into two clusters that accounted for 64.9% of the variance. The PCoA shows a significant separation between individuals according to the social stress experienced (Adonis, *F*_(1,21)_ = 5.22, *p* < 0.009, Figure 4).

## Discussion

Animal sociality is adaptive, which means that being social increases survival and improves reproductive performance (Kikusui et al., 2006; Snyder-Mackler et al., 2020). Positive social relationships serve to buffer against allostatic load imposed by ecological adverse experiences, such as predation risk, resource competition, infanticide, and social isolation (Rukstalis and French, 2005; Kikusui et al., 2006; Brent et al., 2014; Siracusa et al., 2022). Here, we investigated the effects of prolonged chronic social isolation stress (from post-natal and post-weaning into adulthood) on behavioral, cognitive, and physiological performance in social degus. Overall, and in agreement with our predictions, prolonged chronic social isolation led to an increase in anxiety-like behaviors, a decrease in social and working memory, and a decrease in the complexity of the time series of VO_2_ consumption. Interestingly, our results suggest that using MF-DFA on VO_2_ time series could be a useful diagnostic tool for the aging process and neurodegenerative disease (see Zorick et al., 2020 for MF-DFA in EEG signals).

It has always been suggested that more than one test should be used to measure anxiety in order to accurately characterize anxiety-related behavior and to strengthen interpretations (Bailey and Crawley, 2009). These are the main reasons we also used the novel object open field and the light–dark box tests in this study. The classical open field test, which assesses exploration of a novel environment and general locomotor activity, has been employed as an initial screening test for anxiety-related behavior in rodents. No significant differences between treatments were observed in the open field test; however, in the novel object open field test, the CI group behaved differently than the CTRL group. CI degus were less keen to explore the novelty and spent less time in the center of the arena. Similarly, during the light–dark box test, CI males displayed a lower number of transitions (i.e., number of times the animals crossed from the light box to the dark box, and vice versa), and it took them almost three times as long to enter the dark box, suggesting that stressed animals were more insecure. Moreover, CI degus had a longer latency to enter the dark chamber for the first time, suggesting less willing behavior to novelty. Using both tests, we demonstrated high levels of anxiety and hyperreactivity behavior in socially isolated animals. Our results are consistent with previous reports in rats, where anxious, hyperreactive, or hyperaroused rats were less likely to explore novelty, suggesting a deficit in motivation (Rygula et al., 2005; Thorsell et al., 2006). Similar levels of anxiety-like behavior and locomotor hyperactivity have been observed in socially isolated mice and rats (Levine et al., 2007; Medendorp et al., 2018). In addition, anxiety-like behavior using the light–dark box test, specifically the number of light–dark transitions, has been used as an index of activity exploration in mice (Bourin and Hascoët, 2003).

To evaluate how prolonged social isolation stress affects sociability and social novelty preference, we used the three-chamber paradigm. Social recognition memory, which is also necessary for the stability of social groups, reflects the ability to identify and remember conspecifics (Kogan et al., 2000). Using the Recognition Index (RI), we found in session 1 that CI degus had lower values of RI compared to CTRL. These data suggest that degus’ sociability was negatively affected by stress treatment. Both groups showed a clear preference for the compartment containing the unfamiliar conspecific. CTRL and CI animals spent about 35 and 25%, respectively, of their time interacting with the unfamiliar degus, and 5 and 8%, respectively, of their time with the inanimate object. Previous studies demonstrated that chronic stress reduces social motivation and social interaction, particularly in highly anxious animals (Lukkes et al., 2009; Sandi and Haller, 2015; Rivera et al., 2021a). However, the prolonged chronic isolation stress did not diminish social abilities (Sandi and Haller, 2015; Rivera et al., 2021a). As with in humans, stressful situations can promote affiliative behavior or group cohesion to provide a buffer against stress and improve health and well-being (Kikusui et al., 2006; Taylor, 2006; Oppenheimer et al., 2016; Hsiao et al., 2018). In this context, previous studies in stressed rodents have shown a willingness to interact with conspecifics to potentially ameliorate negative emotions for positive neurochemical rewards (Kikusui et al., 2006; Gilles and Polston, 2017). During Session II, the social recognition memory was assessed by the spontaneous approach behavior observed in an individual who was re-exposed to a familiar conspecific. Our results indicated that CI degus had lower RI values compared to CTRL. Moreover, we found that CI males spent almost twice as much time interacting with Partner I compared to CTRL, indicating that prolonged social isolation affects social memory. Our results are in line with the reported long-lasting effects of early life stress in memory performance in other animal models (Ibi et al., 2008; Mumtaz et al., 2018) and even degus (Rivera et al., 2021a).

We used the novel object recognition test to evaluate cognition, particularly working memory, attention, and preference for novelty (Popović et al., 2009). We observed that working episodic memory (measured by RI in the NLR/NOR task) was impaired in male degus under CI treatment. During the NLR session, both groups showed similar time interacting with the moved object; however, CI animals spent almost three times as much time interacting with the familiar object as CTRL degus. Furthermore, during the NOR session, the CI animals spent less than half as much time interacting with the novel object compared to the unstressed animals, and almost twice as much time interacting with the familiar object. These results suggest that CI degus exhibit an impairment of the rodents’ innate preference for novel experiences (such as exploring new objects). Previous works using mice and rats have similarly described deficits of spatial memory resulting from social isolation (Voikar et al., 2005; McLean et al., 2010; Famitafreshi and Karimian, 2018). Moreover, we previously found that chronic isolation affected the cognitive performance in the NLR/NOR task using the same isolation protocol described in this paper (Rivera et al., 2020).

We also investigated the potential effects of social isolation stress on degus’ energy expenditure (e.g., BMR). We predicted that more anxious and hyperactive individuals would have a higher energy expenditure than non-stressed individuals (Mueller and Diamond, 2001). While it is intuitive to expect that organisms that are physically active, bold, and exploratory will acquire and expend energy at higher rates than organisms with opposite behaviors, previous evidence is contradictory (Mueller and Diamond, 2001; Careau et al., 2008). High levels of stress, anxiety, and hyperactivity can put a strain on the sympathetic nervous system, increase cardiac output and respiration, and cause individuals to maintain higher activity levels or muscle tone than calm, relaxed individuals (Gilmour et al., 2005). However, our results did not support the stress and energy expenditure relationship. Social isolation stress had no effect on BMR, which could be attributed to the fact that BMR represents the minimum maintenance cost of organisms and is measured in post-absorptive, non-reproductive, resting. and thermoneutral zone organisms (McNab, 2002). Its estimation took into account a short segment of the metabolic record with the lowest and most stable values (5–10 min after steady state, see Methods). Thus, by focusing only on the steady-state period, BMR estimation excludes fluctuations in metabolic rates that could potentially reflect behavioral syndromes (i.e., hyperactivity, anxiety-like behavior). Body mass also did not vary between treatments, although it has been reported to be a variable strongly influenced by stress levels (Patterson and Abizaid, 2013). While chronic social isolation did not affect BMR, it did affect the multifractality of the VO_2_ time series. Importantly, MF-DFA allowed us to detect anomalies in the regularity of the time series. The negative effects of social isolation stress on cognitive performance were accompanied by the loss of complexity VO_2_ time series with a dominant pattern of long-range correlation, reflected in a lower ∆h and ∆α in the multifractal spectrum. The decrease in complexity observed in oxygen consumption rate, a reliable indicator of metabolic status, could potentially indicate a decrease in the regulation of homeostatic processes (Modell et al., 2015). Previous studies have reported that a reduction in physiological complexity predisposes organisms to frailty syndrome, a manifestation of physiological decline due to decoupling of integrated multimodal networks and disabling control loops, resulting in functional decline (Lipsitz, 2004; Schubert et al., 2009). In our study, the degradation or loss of structural complexity in stressed animals may be associated with an increase in inflammatory markers, making them more susceptible to premature immune aging. Indeed, exposure to adversity associated with traumatic stress events (e.g., war, abuse) in humans appears to accelerate markers of biological aging (Jergović et al., 2014; Lohr et al., 2015) and even makes them more susceptible to acceleration, progression, and worsening of symptoms of neurodegenerative disorders such as Alzheimer’s, Parkinson’s disease, and Huntington’s disease.

Overall, our results reveal that chronic social stress significantly affects anxiety-like behavior, social and working memory, and the fractality of the VO_2_ time series. Likewise, the simultaneous analysis of all variables through PCoA showed a clear segregation of variables into two well-defined clusters, corresponding to healthy and stressed degus. To the best of our knowledge, this is the first study integrating cognitive and behavioral performance with the multifractal dynamics of physiological signals in response to chronic prolonged social stress.

Our results highlight: (1) the importance of incorporating methods, such as MF-DFA, that capture the complex nature of biological systems; and (2) the need to deepen our knowledge of organisms’ response to stress through studies that integrate different levels of organization (from physiology to cognition and behavior). Because stress responses vary among individuals (i.e., sex-dependent manner) and may be determined by social experiences during early development and adulthood (Lukkes et al., 2009; Rivera et al., 2021a), future studies focusing on the cross-generational effects of stress and including other complexity analyses of biological signals will allow us to better predict how social environments affect individuals, populations, and species. This data can then be useful for disease detection and diagnosis (Dutta et al., 2013; Zorick et al., 2020).

## Data availability statement

The original contributions presented in the study are publicly available in Zenodo https://doi.org/10.5281/zenodo.8397447.

## Ethics statement

The animal study was approved by Bioethical and Biosafety Committee of the Faculty of Biological Sciences of the Pontificia Universidad Católica de Chile (CBB-121-2013). The study was conducted in accordance with the local legislation and institutional requirements.

## Author contributions

DR contributed to behavioral formal analysis, writing, reviewing, editing, original draft preparation, funding acquisition, and visualization. GC contributed to physiological formal analysis, writing, reviewing, editing, original draft preparation, and visualization. JB contributed to multifractality formal analysis, writing, and visualization. FB contributed to conceptualization, supervision, and funding acquisition. All authors contributed to the production of the article.

## Footnotes

^1^ http://www.R-project.org/

## In memoriam

We express our deep gratitude to our co-author, FB, who made significant contributions to this project. He passed away before completion of this work and we would like to take this opportunity to honor his memory and valuable contributions. He will be greatly missed.
